# Climate effects on land management and stream nitrogen concentrations in small agricultural catchments in Norway

**DOI:** 10.1007/s13280-020-01359-z

**Published:** 2020-09-12

**Authors:** Hannah Wenng, Marianne Bechmann, Tore Krogstad, Eva Skarbøvik

**Affiliations:** 1 Norwegian Institute for Bioeconomy Research (NIBIO), Norwegian University of Life Science (NMBU), Fredrik A. Dahls vei 20, 1430 Ås, Norway; 2 Faculty of Environmental Science and Natural Resource Management, Norwegian University of Life Science – NMBU, Fougnerbakken 3, 1432 Ås, Norway

**Keywords:** Agricultural management, Climate change, Growing season, Nitrogen leaching, Water quality

## Abstract

Land use and climate change can impact water quality in agricultural catchments. The objectives were to assess long-term monitoring data to quantify changes to the thermal growing season length, investigate farmer adaptations to this and examine these and other factors in relation to total nitrogen and nitrate water concentrations. Data (1991–2017) from seven small Norwegian agricultural catchments were analysed using Mann–Kendall Trend Tests, Pearson correlation and a linear mixed model. The growing season length increased significantly in four of seven catchments. In catchments with cereal production, the increased growing season length corresponded to a reduction in nitrogen concentrations, but there was no such relationship in grassland catchments. In one cereal catchment, a significant correlation was found between the start of sowing and start of the thermal growing season. Understanding the role of the growing season and other factors can provide additional insight into processes and land use choices taking place in agricultural catchments.

## Introduction

The agricultural sector is under pressure to respond to energy and food security challenges and to reduce greenhouse gas emissions. At the same time, the threat of climate change and an increasing demand on bioeconomic products may intensify the pressure on agricultural production (Rosegrant et al. 2013). Furthermore, agricultural production is one of the main sources of elevated nutrient concentrations in water bodies both in Norway and globally (Ulén et al. 2007; Giri and Qiu 2016). The changing climate affects agricultural production systems and also hydrology, and thereby influences nutrient and soil losses (Deelstra et al. 2011; Giri and Qiu 2016).

The Intergovernmental Panel on Climate Change (IPCC) developed different Representative Concentration Pathways (RCP) for climate change research. If the RCP 4.5 (intermediate emissions) is assumed for Norway, the annual average temperature is expected to rise by approximately 2.7 °C (calculated for the period 1971–2000 to 2071–2100), with the greatest change in Northern Norway (Hanssen-Bauer et al. 2015). Moreover, higher temperatures can lead to a longer thermal growing season, here defined as the period when the mean temperature exceeds 5 °C (Ruosteenoja et al. 2011). In Norway, the RCP 4.5 scenario projects an extension of the thermal growing season by one to two months (Hanssen-Bauer et al. 2015). Previously, Jeong et al. (2011) showed an increase of the vegetative growing season (phenology) for the temperate zone in the Northern Hemisphere during the period 1982–2008. Consequently, this may imply earlier timing of agricultural management in spring (e.g. seedbed preparation, sowing), the introduction of new crop varieties adapted to a longer growing season and higher yields (He et al. 2018; Wiréhn 2018). However, it is still unknown if a prolonged thermal growing season has or will have an impact on water quality. Øygarden et al. (2014) and Wiréhn (2018) suggested that a prolonged thermal growing season can reduce the risk of nitrogen (N) leaching due to better utilisation of nutrients and a longer period with vegetation cover. Nevertheless, uncertainties exist since it is not known how agricultural management may adapt to climate change, including the potential for increased N application due to expectations of higher yields. Furthermore, it is uncertain how soil mineral N will change due to higher temperatures (He et al. 2018).

The connection between climate change, growing season length and agricultural production has been discussed by a number of researchers, for example, Børgesen and Olesen (2011); Ruosteenoja et al. (2011), Øygarden et al. (2014) for Northern European Countries and He et al. (2018), Morgounov et al. (2018) for North America. Most of these studies applied a model-based approach and did not use monitoring data that enables a retro-perspective view on this topic.

Therefore, the aim of the present study was to investigate three objectives to determine whether:

1. Climate change has already affected the length of the thermal growing season in the monitored catchments investigated
2. Farmers have adapted their sowing and harvesting dates to this change
3. A prolonged thermal growing season has affected N leaching to streams

The analysis was based on 27 years (1991–2017) of monitoring data from seven small Norwegian agricultural catchments. The data were statistically analysed, using Mann–Kendall Trend Test, Pearson correlation and a linear mixed model.

## Materials and methods

### Study sites

**Fig. 1:**
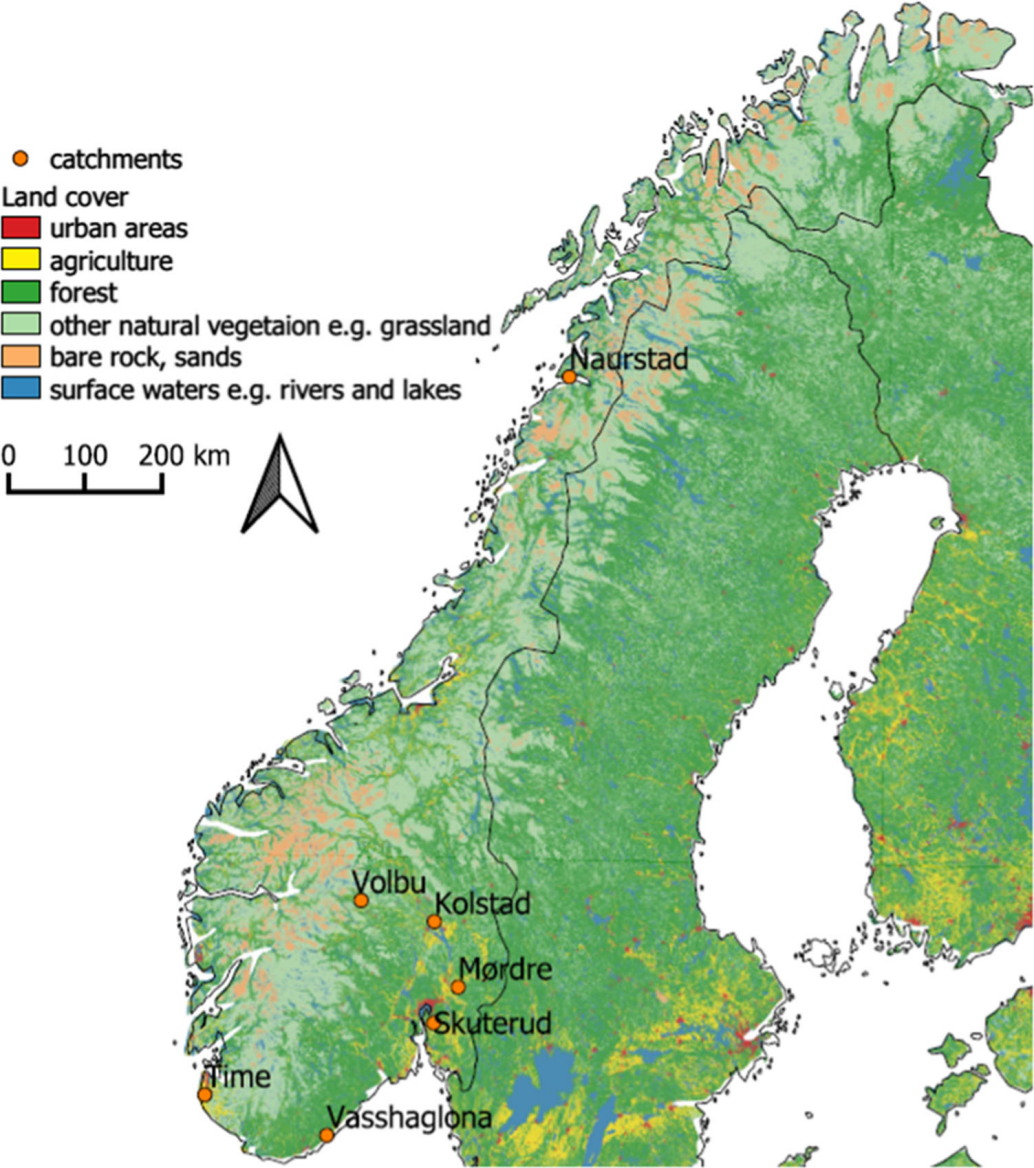
Location of the seven monitored JOVA catchments in Norway. Land use data: CORINE land cover (https://land.copernicus.eu)

Data were used from seven small agricultural catchments (87 to 680 ha), covering different regions of Norway (Fig. 1). The catchments belong to the long-term Norwegian Agriculture Environmental Monitoring Programme (JOVA), which has been run by the Norwegian Institute of Bioeconomy Research since 1991. The widespread network, with monitoring stations located at the outlet of each catchment, made it possible to represent different Norwegian climate zones, soils, topography and elevation, and therefore also different agricultural production systems such as cereal, grass and vegetable production (Tables 1 and 2).

**Table 1:** Main characteristics of the monitored JOVA catchments

Catchment	Total area (ha)	Agricultural land use (%)	Main crops	Soil texture	Elevation range (m.a.s.l.)	30-year normal *T* (°C)	Monitoring period
Skuterud	450	62	Cereals	Silty clay, loam, silty loam	91–146	5.3	1994–2017
Mørdre	680	65	Cereals	Silt, silty clay, loam	130–230	4.0	1992–2017
Kolstad	310	68	Cereals	Loam, loamy sand	200–318	3.6	1991–2017
Time	97	88	Grass	Loamy sand, organic	35–100	7.2	1996–2017
Naurstad	146	42	Grass	Peat soil	4–91	4.5	1994–2017
Vasshaglona	87	48	Vegetables, potatoes, cereals	Sand, loam	5–40	6.9	1998–2017
Volbu	166	43	Grass	Silty sand, silty loam	440–863	1.6	1994–2017

**Table 2:** For the monitoring period: annual mean temperature (*T*) in °C, annual sum of precipitation (P) in mm, mean thermal growing season length in days per year, annual (1st May to 1st May) flow-weighted TN and NO_3_-N concentrations in mg l^−1^, annual N fertiliser input in kg ha^−1^ and annual (1st May to 1st May) mean N balance (surplus) in kg ha^−1^

Catchment	*T* (°C)*	P (mm)*	Growing season length (days)*	TN (mg l^−1^)	NO_3_-N (mg l^−1^)	Fertiliser input (kg ha^−1)^*	N balance (kg ha^−1^)*
Skuterud	6.6+	824	200+++	5.8	4.5	163	6.0
Mørdre	6.1++	709	184+	5.0	3.6	126	5.3−
Kolstad	4.9++	734	172	10.9	9.3	159	6.5
Time	8.3	1282+	243+++	6.5	4.6	375	10.3+++
Naurstad	5.5+++	1278	179+	1.1	0.4	104−	2.9−
Vasshaglona	8.4	1459	230	5.8	4.5	188	8.81
Volbu	3.1	613++	155	3.2	2.4	105−	2.8−

*Significant levels: +/– 0.05 > *p* > 0.01; ++/– 0.01 > *p* > 0.005; +++/—0.005 > *p*; and trend direction is marked with + for upward and − for downward

The catchments represent the main agricultural production systems of their specific region: extensive grass production in the north and in the mountains (Naurstad and Volbu); intensive dairy production in western Norway (Time); a mix of dairy and cereal production in inland southern Norway (Kolstad); cereal production in the south-eastern part of the country (Skuterud, Mørdre); and vegetable and cereal production in southern Norway (Vasshaglona) (Fig. 1). The cereal production areas are found in regions where there is relatively little precipitation during the harvest period (Bechmann 2014). The production systems are reflected in fertiliser input and water quality. For example, Time and Vasshaglona show a high fertiliser input and high TN concentrations in the streams due to intensive use of grassland for meat and dairy production and intensive vegetable production, respectively. All catchments have a widespread drainage system. The climatic variation can be seen in the runoff and in the thermal growing season length (Table 2).

### Monitoring data

The analysis was based on 27 years of observation data on hydrology, N concentrations and agricultural management. The earliest time series started in 1991 and ended in 2017 (Table 1). Water level was measured continuously at catchment outlet streams, using a pressure transducer combined with a Campbell data logger, and converted to discharge (flow) at standard weirs. The data logger controlled the rate of automatic water-sampling and these sub-samples were combined as composite samples on a volume proportional (flow-weighted) basis and collected every 14th day (Deelstra et al. 2013). Annual and monthly flow-weighted concentrations were calculated by summarising daily loss over a month or a year and divided by total runoff during the corresponding period. Daily loss was calculated as daily runoff multiplied by N concentrations in the corresponding fortnightly water sample. This study used the annual period from 1st May to 1st May (agro-hydrological year) to account for the time lags between agricultural management and weather impacts in the catchment. In this way, the thermal growing season of one calendar year will relate to N concentrations the following autumn, winter and spring. The analytical method used to determine TN and NO_3_-N concentrations involved oxidative digestion with peroxydisulfate, which is a colorimetric method (Norwegian Standard ISO 11905-1:1997). Since the 1990 s, information about farm management has been collected on a yearly basis for each individual field. Farmers have provided information about crop type, sowing and harvesting dates, type and date of tillage, yield, amount of applied fertiliser (mineral and manure), type and number of animals, and amount and date of applied pesticides (Bechmann 2014). Data on temperature and precipitation were recorded by local weather stations located in or close to the catchments. The thermal growing season length was calculated based on daily average temperature. The start of the thermal growing season was defined as the day on which the daily average temperature remained higher than 5 °C after seven days, and the end was defined as the day when the daily average temperature had been lower than 5 °C after seven days (Carter 1998; Hanssen-Bauer et al. 2015). The actual agricultural growing season for cereal crops was defined as the period between sowing (first day) and harvesting (last day) of crops, which reflects the farmers’ actual management of the field (Waha et al. 2012). We used both the first day of sowing and the day when 50% of the area was sown to correlate it with thermal growing season length. Nitrogen balances were based on the agricultural management data, calculated as applied N in fertiliser and manure, minus N removed by yield (the latter was based on farmers’ estimates of yield and standard values for N content in the product) (Bechmann 2014).

### Statistical analysis

The following statistical tests were performed: Mann–Kendall Trend Test, Pearson correlation and a linear mixed effects model.

The Mann–Kendall non-parametric trend test can account for the non-normality of hydrological data (Yue et al. 2002). The test was applied to see if there were long-term changes for the following parameters: thermal and actual agricultural growing season length, flow-weighted TN and NO_3_-N concentrations, N input, N balance, yield, harvest and sow dates, stream discharge, precipitation and temperature. The Mann–Kendall tests were based on monthly and annual data, using calendar years and agro-hydrological years.

The linear mixed effects model provides a technique for analysing the water quality data on the basis of non-probabilistic sampling (Lessels and Bishop 2013; Giri and Qiu 2016). The model was not used as a prediction tool, but to help explain processes. The linear mixed effects model considered both fixed effects and random effects on the response variables TN and NO_3_-N concentrations. We chose N concentrations and not fluxes, because concentrations are less dependent on runoff, and may therefore be better suited to identify other effects such as thermal growing season length, and variables such as temperature, discharge and or agricultural practices (Bechmann 2014). The fixed effects consider global effects, whereas the random effects consider the individuality of each catchment. Furthermore, linear mixed effects models can deal with dependency in observations and different spatial and temporal scales. Monitoring is based on repeated measurements on the same individual, in our case the stations in the chosen catchments. The statistical design is a parallel group design. The intention was to go beyond the chosen catchments and give more general assessments. The model, which was performed with R version 3.5.2, is described below:

- Linear Mixed Model (LMM) describes log TN and log NO_3_-N as a function of growing season length, fertiliser input, N balance, discharge and temperature. It was applied to study whether the thermal growing season, fertiliser input, N balance, discharge and temperature (representing climate), has an impact on the TN and NO_3_-N concentrations in the streams for all catchments.

Precipitation was omitted from the LMM on the assumption that it provides the same information as the discharge variable (Øygarden et al. 2014). Years with incomplete observations for all variables were taken out of the analysis. In total, there were 158 observations over a period of 16–27 years for all catchments. The water quality concentrations of TN and NO_3_-N were log-transformed to a normal distribution. The LMM was applied to three types of datasets: (1) the aggregated dataset; (2) the data in four catchments with cereal production (96 observations); and (3) data in three catchments with grass production (62 observations). Pearson correlation was applied to view the catchments individually. The statistical significance level was set at 5% and a (non-significant) tendency to change at 5–10% following the method by Skarbøvik et al. (2014).

## Results and discussion

### Change in the thermal growing season length

**Fig. 2:**
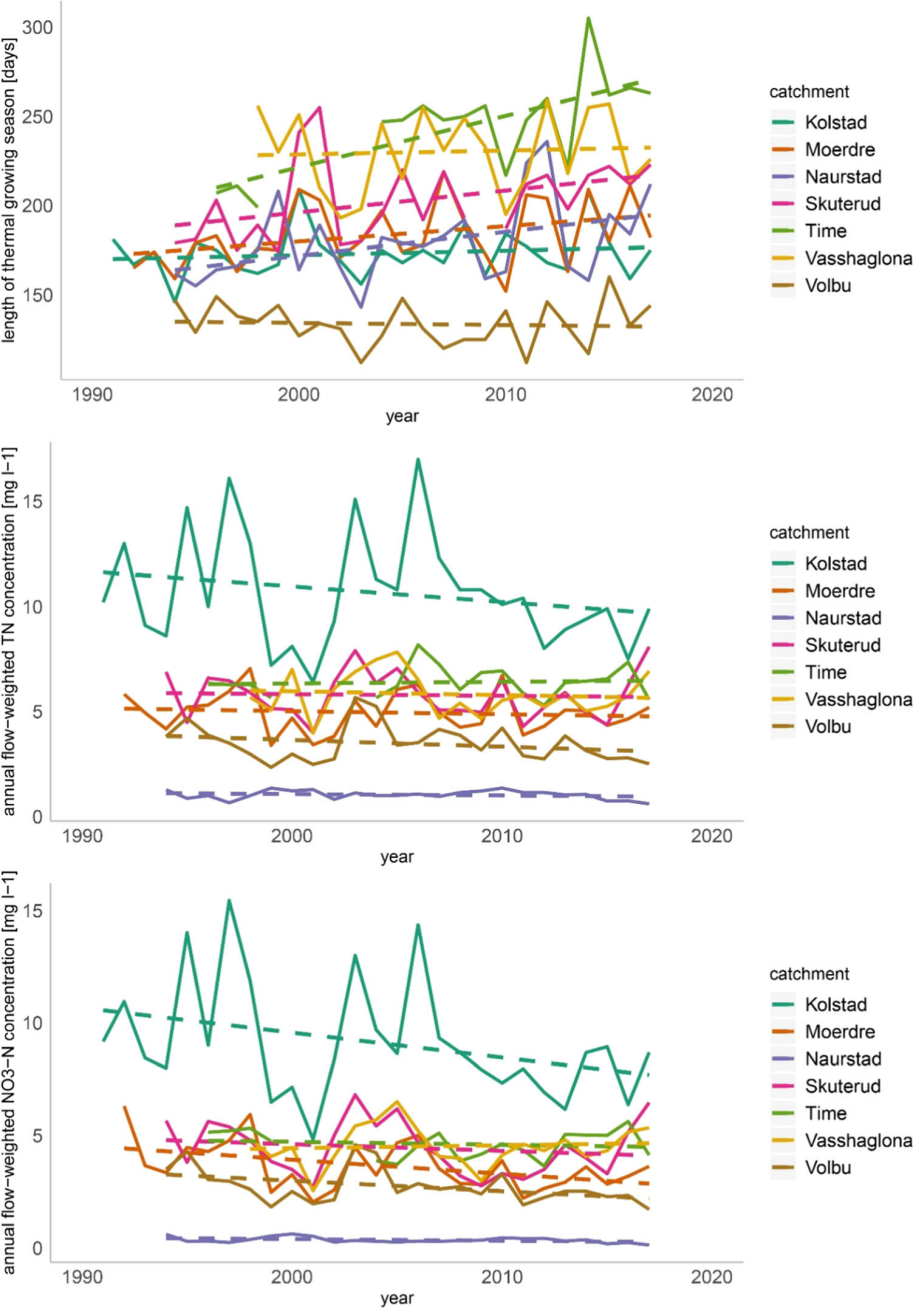
Change in thermal growing season length (**a**), annual flow-weighted TN concentration (**b**), and annual flow-weighted NO_3_-N concentration (**c**) for the seven analysed catchments. The dashed lines illustrate the linear long-term changes and are not related to statistical significance

The Mann–Kendall Trend Test of the aggregated data for all seven catchments showed a significant increase of the thermal growing season length with an average change of 0.66 days per year. When analysing the catchments separately, four of seven showed a significant increasing trend in thermal growing season (Fig. 2a): Skuterud, Mørdre, Naurstad and Time (Table 2). The three remaining catchments Kolstad, Volbu and Vasshaglona showed no significant trends.

The Mann–Kendall Trend Test showed that four catchments had significant increases of the annual mean temperature (Table 2) and in addition the catchment Time indicated to increase (*p* > 0.09). The Mann–Kendall test of the seasonal changes of mean air temperature showed that six out of seven catchments had a significant increase in monthly mean temperature either in March, April or May, and three out of seven catchments saw a significant increase in the average monthly air temperature during autumn (September). This increase in spring and autumn temperatures was also shown for the periods 1985 to 2014 and 1971 to 2000 by Hanssen-Bauer et al. (2015).

**Fig. 3:**
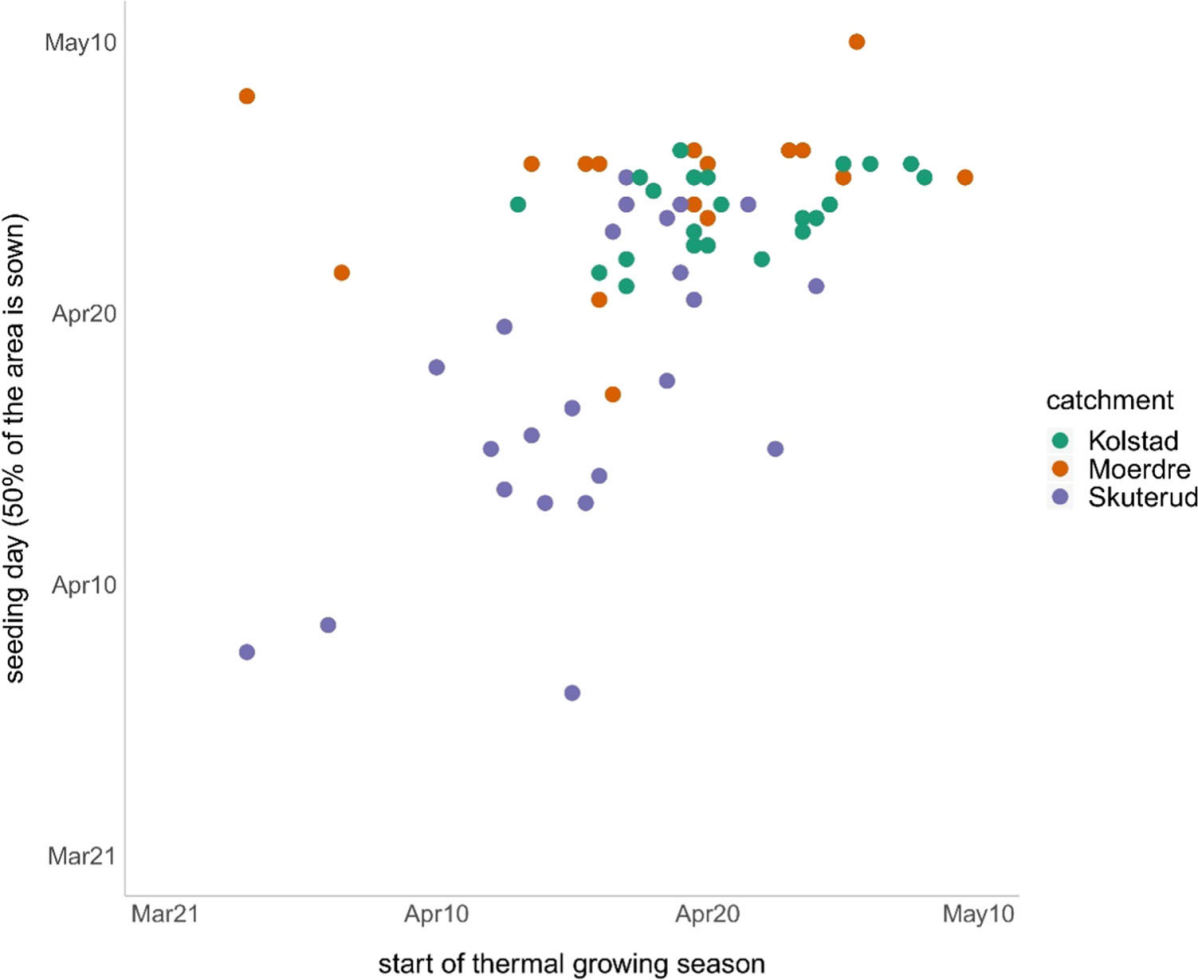
Correlation between the first day of sowing of spring cereals and the start of the thermal growing season. Pearson correlation: Skuterud 0.63

Warmer spring temperatures accelerates the phenological development of plants (Menzel et al. 2006; Jeong et al. 2011) and a change in thermal growing season may affect the actual agricultural growing season and management (Børgesen and Olesen 2011; Ruosteenoja et al. 2011; He et al. 2018). This would mean earlier sowing and, if no change in plant varieties occur, earlier harvesting of spring cereals. A possible shift in the varieties of cereals used to those better adapted to a longer growing season and with higher yield potential could result in later harvesting (Seehusen et al. 2015; He et al. 2018). Further, with shifting the sowing date earlier in the year and with simultaneous increased CO_2_ concentrations and precipitation an increase of yield could be expected as He et al. (2018) simulated for a Canadian region. For Skuterud, Mørdre and Kolstad, which are cereal production catchments, long-term changes for the first day of sowing and last day of harvesting were analysed. No significant changes over time could be found. In Skuterud there was a significant Pearson correlation (coeff. 0.63) between start of the thermal growing season and the day when 50% of the area is sown (Fig. 3). Considering the case, when at least one farmer had started to sow, there was a significant Pearson correlation with the coefficients 0.62 for Skuterud and 0.42 for Mørdre. Kolstad showed no significance (coeff. 0.18). Here, the extreme conditions show what is possible and that there are farmers which will likely change their sowing date of spring cereals in accordance with changes in spring temperatures due to interannual climate variability. It provides a scenario of farmers’ adapting their sowing activities to a changing growing season over time. Other authors have found a weak relationship between spring temperature and farmers’ sowing dates due to e.g. the number of frost days (van Oort et al. 2012).

Moreover a prolonged thermal growing season will not always lead to earlier sowing, because other factors also play a role in the farmers’ decision-making processes. Kolberg et al. (2019), for example, found that the most limiting factor for early plant development in Norway is soil moisture, because of its impact on soil strength, trafficability and aeration. Riley (2016) argued that soil water content is the main factor in Norwegian farmers’ decision to sow or harvest. Soil moisture was also found to be an important factor for agricultural sites in the Canadian prairies (Bootsma and De Jong 1988). More precipitation in spring and autumn is expected, which will affect the workability of the soil at the time the cereals are sown in Norway (Kolberg et al. 2019). Soil type, weather forecasts, available working days, workforce and machinery also play a role in the decision-making process (Waha et al. 2012; Kolberg et al. 2019). Farmers choose suitable cropping periods to optimise production on the basis of these factors (Waha et al. 2012). The current length of the growing season is limited by low temperatures, soil moisture in spring and autumn and the availability of solar radiation in northern countries. These factors also limit the productivity of crops (Olesen and Bindi 2002; Seehusen et al. 2015). Here, the trend for the annual average cereal yields (including spring and winter wheat, oat and barley) was analysed for Skuterud, Mørdre, and Kolstad. The Mørdre and Kolstad catchments indicated an upward tendency in cereal yields (*p* = 0.08, *p* = 0.1). He et al. (2012) saw a large potential for increasing yields of spring wheat in Canada due to earlier seeding dates, driven by increase in temperature and increase in precipitation during important growth stages. However, this is in contradiction with Seehusen et al. (2015), who argued that the trend in Norway is rather a stagnation in cereal yields due to poor physical soil conditions and poor drainage systems, reinforced by weather conditions. Other limiting factors for the yield can be warmer temperatures and less precipitation during the growing season (He et al. 2018).

### Trends in water discharge, TN and NO_3_-N concentrations

For the Nordic countries, an increase of annual precipitation is predicted (Hanssen-Bauer et al. 2015; Wiréhn 2018), hence a consideration of the potential for changes in stream discharge. Two out of seven catchments showed a tendency in increasing annual water discharge (0.07 < *p* < 0.1). Skuterud had a significant upward trend. In terms of the annual TN concentrations no significant trend could be found. For the annual NO_3_ -N concentrations, no significant trends could be found, and only Kolstad indicated a downward trend (*p* > 0.08). It might be considered, however, that interannual changes might weaken signals for significant trends (Table 2, Fig. 2b, c).

The explanation for the changes in N concentrations in these small catchments may differ from catchment to catchment. For Mørdre and Volbu, the N balance decreased significantly during the monitoring period until 2017, which contributes to the decrease in N concentrations (Valkama et al. 2013). In Time, there was an increase in application of mineral fertiliser and the N balance also showed a significant upward trend (Table 2). In Kolstad, an increase in grassland and decrease in cereal area can be expected to contribute to a decrease in nitrogen concentration. In addition, increasing discharge often leads to a dilution of N concentrations. Such a dilution effect for NO_3_-N has been shown by Bieroza et al. (2018) based on a high-frequency dataset from an agriculturally dominated catchment in the UK.

### Trends in agricultural management

The Mann–Kendall analysis on fertiliser input revealed significant upward trends for the total input as well as for mineral fertiliser. The analysis of total fertiliser input for each single catchment indicates an upward trend for Kolstad and Time (*p* < 0.1). Significant downward trends in total fertiliser input and mineral N application could be observed in Naurstad and Volbu (Table 2). This is probably due to the increasingly extensive grass and animal production i.e. less animals per ha and intensity of management.

For manure application, Kolstad and Vasshaglona showed a significant upward trend. In Kolstad, there is an ongoing change from cereal production combined with animal husbandry, to more animal husbandry and grass production. The significant upward trends in manure application in Vasshaglona can be linked to a probable intensified production of vegetable and potato and less cereal production (e.g. Bechmann et al. 2008). Correspondingly, an intensification of the production in the Time catchment (dairy and grass production) is a probable reason for the significant upward trend in the application of mineral fertiliser in this catchment. Changes in mineral N application reflect changes in cropping systems, whereas the change of N application in the form of manure reflects changes in dairy, meat and grass production (Zimmermann et al. 2017).

### Effect of thermal growing season, climate and nitrogen input on nitrogen concentrations

The results of LMM applied on the aggregated data show a significant relationship between thermal growing season length and TN and NO_3_-N concentrations in the streams (Table 3). Furthermore, for cereal production systems (4 catchments), the thermal growing season length played a significant role in reducing TN and NO_3_-N concentrations (Table 3). Five out of seven catchments showed a negative Pearson correlation between TN concentrations and growing season length (Tab 4). However, Skuterud and Mørdre (cereal catchments) showed a significant negative correlation between TN concentrations and growing season length (Table 4). The grass production systems behave differently since the growing season length had no significant effect on the TN and NO_3_-N concentration in the streams (Tables 3, 4).

**Table 3:** Results of the linear mixed effects model. The significance level is 5%, and the slope gives the direction (negative is downward, positive is upward) and the magnitude of the relationship, the bold fonts depict significant *p* values

Dataset	Fixed effects	Constituent	Growing season	Total N fertiliser input	Discharge	Average air temperature	N balance
All catchments	Slope	TN	− 0.002	< 0.001	<− 0.001	0.01	0.02
		NO_3_-N	− 0.003	0.001	<− 0.001	0.03	0.02
	*p* value	TN	**0.002**	0.3	**< 0.001**	0.5	**0.05**
		NO_3_-N	**0.014**	0.3	**0.03**	0.2	**0.04**
Cereal production systems	Slope	TN	− 0.004	0.001	<− 0.001	0.03	0.02
		NO_3_-N	− 0.006	< 0.001	<− 0.001	0.05	0.03
	*p* value	TN	**<0.001**	0.3	**0.003**	0.1	**0.01**
		NO_3_-N	**<0.0014**	0.6	**< 0.001**	**0.01**	**0.001**
Grass production systems	Slope	TN	<0.001	< 0.001	<− 0.001	− 0.01	0.004
		NO_3_-N	<0.001	0.002	< 0.001	0.005	− 0.003
	*p* value	TN	0.8	0.5	0.2	0.7	0.8
		NO_3_-N	0.7	0.3	0.9	0.9	0.9

**Table 4:** Pearson correlation coefficient between total nitrogen and different variables for each catchment

	TN–growing season length	TN–precipitation	TN–discharge	TN–temperature	TN–N balance
Kolstad (cereal, grass)	− 0.2	− 0.4*	− 0.4*	0.1	0.3
Mørdre (cereal)	− 0.5**	− 0.6**	− 0.5**	− 0.07	− 0.09
Naurstad (grass)	0.2	− 0.3	− 0.5*	− 0.2	0.4
Skuterud (cereal)	− 0.5*	− 0.4*	− 0.6**	− 0.02	0.2
Time (grass)	0.3	− 0.1	0.1	0.5	0.04
Vasshaglona (cereal, vegetables)	− 0.2	− 0.3	− 0.4	− 0.03	0.2
Volbu (grass)	− 0.2	− 0.3	− 0.1	− 0.2	0.06

*Significant levels: *0.05 > *p* > 0.01; **0.01 > *p* > 0.005; ***0.005 > *p*

**Fig. 4:**
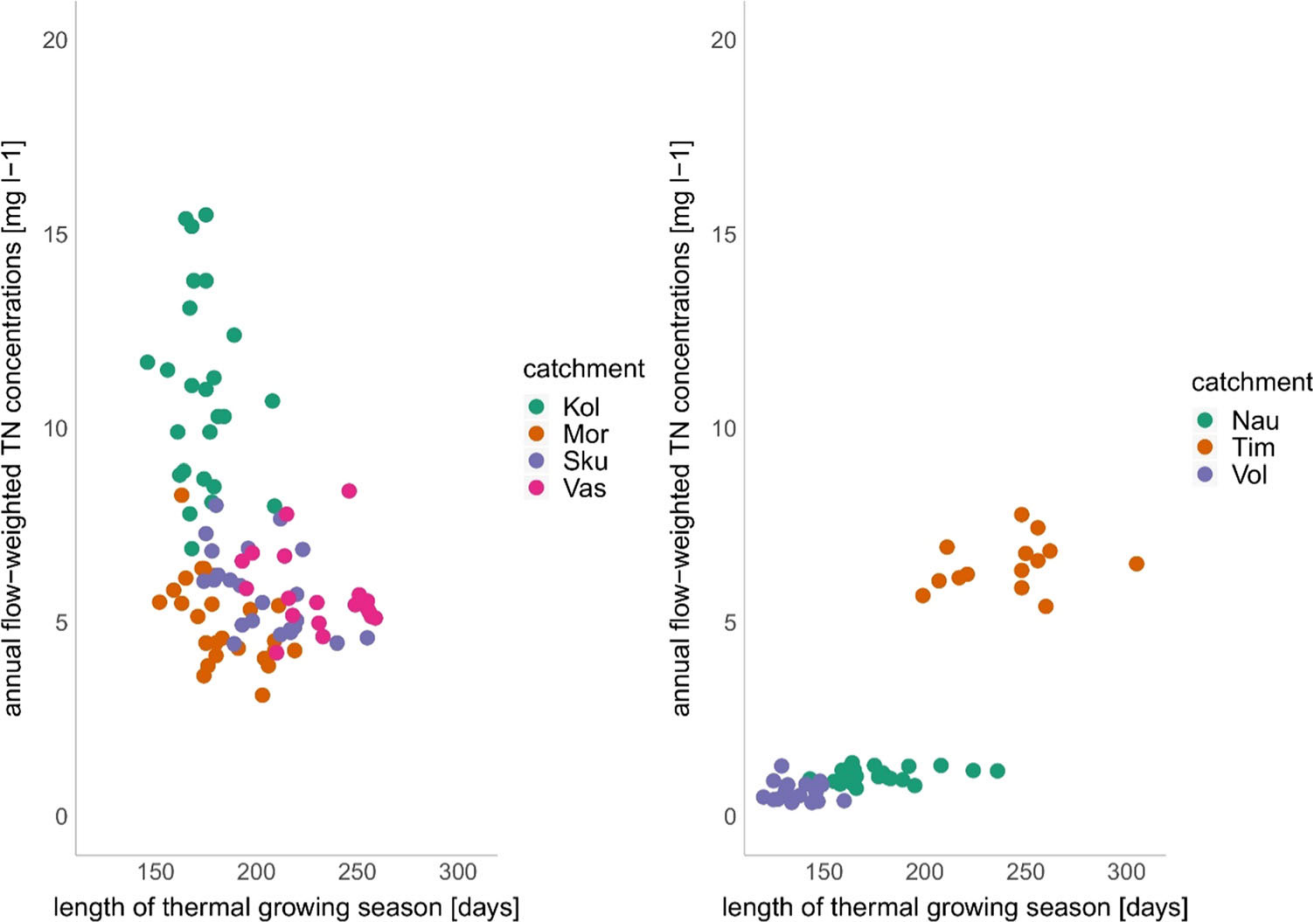
Scatterplot between TN concentration and growing season length for cereal (left) and grass (right) production systems

Additionally, there could be co-variances with the specific catchment properties, such as climate, soil type and intensity of the agricultural production. With only a few catchments in each group, there may be some inherent variables that explain the variation in N concentration between catchments. This can be seen, for instance, in the Time catchment, where the high intensity in grass production corresponds to high N concentrations, compared to the extensive production systems in Naurstad and Volbu (Fig. 4). Furthermore, differences in soil type may affect N concentrations. Soils dominated by coarse texture as in Kolstad are prone to higher soil percolation rates and hence, tend to have higher N concentrations compared to soils dominated by surface runoff which can be found in Skuterud and Mørdre (Table 2) (Bechmann 2014).

The results of the LMM applied to the aggregated data showed also that both the N balance and water discharge play a significant role in regulating nitrogen concentrations (Table 3). Water discharge affects N concentrations in stream water, e.g. by dilution of the concentration during high water discharges (Bechmann 2014, Bieroza et al. 2018). This offers an explanation to the negative slope and correlation in Tables 3 and 4, respectively. In the cereal catchments, discharge showed a significant dilution effect (Tables 3, 4).

The N balance is an indicator of how much N is available in the agricultural soils for leaching (Valkama et al. 2013). When the N balance is positive, there is a risk of more N being leached (Cherry et al. 2008; Valkama et al. 2013). Our results show that N balance has a significant effect in increasing TN and NO_3_-N concentrations for the aggregated data and for the cereal catchments (Table 3). In a study of 14 Nordic time series, Bechmann et al. (2014) also showed a positive significant correlation between N balance and N concentrations.TN and NO_3_-N concentration in the streams increased in line with total fertiliser input in both grass and cereal dominated catchments, although not statistically significant. That means in real terms as fertiliser would increase, so too would TN and NO_3_-N increase in the catchment stream.

Warmer temperatures increase the turnover rate of organic matter, which supports mineralisation of N and might cause an additional risk of N leaching (Patil et al. 2010; Børgesen and Olesen 2011; He et al. 2018). The results of the LMM showed a positive slope for temperature, although not significant. A constraint in terms of the positive effect of growing season length is the availability of light. Even if the spring and autumn gets warmer, and the risk of late spring and early autumn frosts decreases, the availability of light still determines plant development and growth in northern countries (Olesen and Bindi 2002). Although the impact of climate change will positively affect agricultural productivity in northern countries by increasing the resource use efficiency of crops, the negative impacts should not be neglected (Olesen and Bindi 2002; He et al. 2018). Intensification and other land use changes could lead to an increased demand for fertiliser to gain higher yields (Zimmermann et al. 2017), and therefore increasing the risk of N leaching as He et al. (2018) simulated for a Canadian region. Additionally, a warmer climate and prolonged thermal growing season can make regions located further north and at higher altitudes suitable for cereal production (Ruosteenoja et al. 2011; Seehusen et al. 2015), which might also lead to an increased area under agricultural land use.

At the same time parts of northern countries, such as the regions where the Vasshaglona catchment is located, will be under an increased risk of summer droughts (Trnka et al. 2011). This limits the ability of plants to take up nitrogen if no irrigation is available, thereby increasing the risk of N leaching and a decrease in yield (He et al. 2018).

The analysis indicates that not all farmers have adapted their management to a change in the thermal growing season. Therefore, further studies are needed to look at triggering factors and turning points that apply to changes in farmers’ behaviour and agricultural management. Understanding farmers’ perceptions can provide important information to agricultural policy makers. Juhola et al. (2017) undertook an empirical study on farmers’ perceptions of climate change and their vulnerability in Finland and Sweden. Among several positive effects, the prolonged growing season was mentioned by the interviewed farmers and advisors, because it provides a chance to cultivate new crop varieties and could result in higher yields (Juhola et al. 2017). Nevertheless, agricultural policy may have a higher impact on farmers’ behaviour than climate change (Juhola et al. 2017). Grise and Kulshreshtha (2016) showed for a Canadian region that prices, policy and land characteristics played a major role for crop choices. In the long term, Zimmermann et al. (2017) predicted that technology and breeding potential will have a higher impact on farm management and yield than climate change. Agricultural policy and technology development could therefore also affect bioeconomic production and, in turn, water quality.

## Conclusions

In this study, the relationships between climate (thermal growing season), land management (farmers’ activities) and nitrogen concentrations were investigated in seven small agricultural catchments across Norway. The results can be summarised as follows:

- For the first objective, the study found that climate change has affected the length of the thermal growing season, there was an increase in the thermal growing season length in four of the seven catchments, located in different parts of Norway; the south-east (Skuterud, Mørdre), the south-west (Time) and the north (Naurstad).
- Considering objective (2), that farmers have adapted their sowing and harvesting dates to this change, the results were not definite. In two of the south-eastern catchments with cereal production, there was a significant correlation between the start of the growing season and the first day of sowing spring cereals (when at least one farmer had sown), which may indicate that farmers have adapted their agricultural management to changes in the spring temperature. No catchment showed a significant long-term change in sowing dates (considering the first day) for spring cereals, probably because of factors such as soil moisture and trafficability.
- For objective (3), the analysis found that a prolonged thermal growing season has affected N leaching to streams differently for cereal and grass dominated catchments. There was a negative correlation between N concentrations and the length of the growing season in catchments with cereal production, whereas the effect of a prolonged growing season on water quality seems to be limited for catchments with grassland, possibly because of increased fertiliser input, changes in precipitation, temperature, discharge patterns and permanent vegetation cover.

For the future bioeconomy, it will be important to improve understanding of how policy and climate change affect farmers’ activities, catchment processes and resulting water quality. Hence, there is a continued requirement for long-term data-series on water quality, thermal growing season, agricultural management and land use change. In this context, it would also be desirable to define a growing season not only based on temperature, but also on soil moisture or the number of precipitation days before sowing. This would provide a more satisfactory link between this concept and actual agricultural practices. Although the future cannot be absolutely predicted, two main factors may change the agricultural landscape, viz. climate change and a transition to a bioeconomy. The former may affect the growing season and farmers’ choices and opportunities, the latter may change the need for biomass and agricultural products. Hence, both changes may affect runoff and losses of nutrients to agricultural streams and enhance eutrophication processes in water systems. Preparedness is important and in addition to modelling, the continuation of long time-series data gathering is imperative.
